# Supplementary material for: On the Use of Human Mobility Proxies for Modeling Epidemics
Source: PLoS Comput Biol. 2014 Jul 10;10(7):e1003716. doi: 10.1371/journal.pcbi.1003716 (PMC4091706; doi:10.1371/journal.pcbi.1003716)
Supplement: Text S1 — The file contains: additional information on data sources (Section 1). Additional results for lower geographic resolutions and epidemic peak times (Section 2). Sensitivity analysis on cross-border commuting, refined definitions of workplace and residence, refined normalization of phone data (Section 3). Additional details on the simulation algorithm (Section 4). (PDF) [file pcbi.1003716.s002.pdf]

Supporting Information Text S1
On the use of human mobility proxies for modeling epidemics

Michele Tizzoni[1], Paolo Bajardi[2], Adeline Decuyper[3], Guillaume Kon Kam King[4], Christian M. Schneider[5], Vincent Blondel[3], Zbigniew Smoreda[6], Marta C. Gonzalez[5,7], Vittoria Colizza[8,9,10*]

1 Computational Epidemiology Laboratory, Institute for Scientific Interchange (ISI), Torino, Italy
2 GECO - Computational Epidemiology Group, Department of Veterinary Sciences, University of Torino
3 ICTEAM Institute, Université Catholique de Louvain, Belgium
4 CNRS, UMR5558, F-69622 Villeurbanne, France
5 Department of Civil and Environmental Engineering, Massachusetts Institute of Technology, 77 Massachusetts Avenue, Cambridge, MA 02139, USA
6 Sociology and Economics of Networks and Services Department, Orange Labs, France
7 Engineering Systems Division, Massachusetts Institute of Technology, 77 Massachusetts Avenue, Cambridge, MA 02139, USA
8 INSERM, U707, Paris, France
9 UPMC Université Paris 06, Faculté de Médecine Pierre et Marie Curie, UMR S 707, Paris, France
10 Institute for Scientific Interchange (ISI), Torino, Italy
*Email: vittoria.colizza@inserm.fr

1 Data

1.1 Census surveys

Portugal. Data for Portugal are extracted from the database of the National Institute of Statistics (INE, http://www.ine.pt) and refer to the 2001 National Census Survey. The Portuguese National Census Survey is conducted every 10 years and collects a wide range of statistical information about the population of Portugal. The census questionnaire is formed by two parts: one for the household and one for each member of the household. In the latter, each member of the household older than 6 is asked to provide the address of the location where he/she works or studies, if different from the usual place of residence.

Spain. Data for Spain are extracted from the database of the National Institute of Statistics (INE) for the year 2005. More specifically, the INE collects data about the national workforce through a survey called Encuesta de Poblacin Activa (INE, http://www.ine.es/inebaseDYN/epa30308/epa_inicio.htm). The survey is conducted by phone or face-to-face interviews every 3 months over a population sample of 65,000 families, corresponding to about 200,000 people, chosen to be representative of the whole country population. Each family sample is divided in six subsamples and each subsample is replaced every 3 months, so that a single family participates to the survey only for six consecutive quarters of year. The survey asks each family member,

aged 16 or older, his or her work status (employed, active, unemployed or inactive) and, if employed, where is located his/her workplace. The location is reported at the level of province. We downloaded from the INE website the 4 full datasets of the 2005 survey. Each dataset contains about 150,000 individual answers to the questionnaire (available at: `http://www.ine.es/daco/daco42/daco4211/epacues05.pdf`). We extracted from each dataset a commuting network between the Spanish provinces, then, we created a cumulative network, summing the number of commuters on each network connection over the 4 datasets. This procedure assumes that the sample of the survey is different for each dataset, which is not true. However, the census database does not allow to identify the fraction of the sample that recurs in every dataset, therefore the cumulative network over the 4 datasets represents the best approximation of the real commuting network. Eventually, we rescaled the number of commuters traveling on each connection by a factor that takes into account the sampling bias of the survey at the province level.

France. Data for France are extracted from the database of the French National Institute of Statistics and Economic Studies (INSEE, `http://www.recensement-2007.insee.fr/`) for the year 2007. Commuting data are collected each year through a nation wide census survey, which samples all the residents in municipalities with less than 10,000 inhabitants and about 8% of the households in the other municipalities. Then, a full database is generated by assembling 5 surveys conducted on 5 consecutive years, resulting in an overall coverage of about 40% of the population in municipalities with more than 10,000 inhabitants. The final commuting network represents the number of daily commuters between any two municipalities (Communes) of France for work or study reasons. Every individual older than 3 is considered as a student, and tracked by the survey.

Temporal trends. Commuting data for France are collected each year since 2006 thus allowing to explore growing trends over time. We examined the census commuting networks of France reported between years 2006 and 2009 and found that the total number of commuters grew by roughly 1% or less each year, leading to an overall growth of 3.2% over the full period (see Table S1).

year	total number of commuters	yearly change (%)
2006	21,374,056	–
2007	21,690,886	1.5
2008	21,904,484	1.0
2009	22,054,707	0.7

Table S1: Number of commuters in France between 2006 and 2009. The yearly change is calculated with respect to the previous year.

1.2 Mobile phones

Mobile phone data used are standard Call Detail Records (CDR). The CDRs are mobile phone logs collected for billing purposes, where location information (cell id) is generated at the start

of a communication event. The CDR contains the timestamp, call duration and type of events (voice, SMS), as well as the code of the cell in which the communication started. The location data (cell id) have to be decoded to obtain a geographical position. In our case, we used the cell tower geographical coordinates, i.e. the site location where several (directional or omni-directional) antennas can be placed. An antenna covers a specific geographical area depending on the location and population density. In low traffic locations such as rural areas, the antennas will tend to be omni-directional covering a large circular area around the cell tower. In urban areas, three antennas will usually share a high site covering smaller areas. Each site will have directional antennas and cover a 120° arc away from the cell tower. In this study we use information on tower positions, so several cell id can have the same geographical coordinates. All the CDRs were anonymized by the service provider before being transferred to the research team.

Portugal. Phone data for Portugal are extracted from a set of Call Detail Records (CDR) of over 1 million mobile phone users (1,058,197) collected over 12 months between April 2006 and March 2007. The commuting network at the cell site scale consists of 2,068 towers and 232,956 directed weighted edges.

Spain. Phone data for Spain are extracted from the CDRs of 1,034,430 users collected over 3 months of activity between November 2007 and January 2008. The commuting network at the cell site scale consists of 9,788 towers and 354,909 directed weighted edges.

France. Phone data for France are extracted from the CDRs of 5,695,974 users, collected between September 1st and October 15, 2007 and based on the 18,461 towers that cover the whole country.

1.3 Mapping mobile phone records to the resolution of administrative census units

In order to compare the commuting patterns extracted from census and mobile phones data, we coarse-grained the commuting networks at the cell site scale to the resolution of the administrative subdivisions in each country. As an example, Figure S1 shows a map of the mobile phone tower cells in Portugal, along with the boundaries of the Portuguese municipalities, the concelhos.

The land area covered by a cell may largely vary, depending on the characteristics of the municipality: metropolitan areas are usually characterized by a large number of cells, with small sizes, while rural areas can be covered by one cell only, with an area of several squared kilometers. This implies that some of the tower cells are completely included in a single municipality, but others are split in two or more parts among neighboring administrative units.

In order to take into account such heterogeneity, we adopt the following synchronization procedure. We intersect the ArcGIS shapefile of the cells with the one of the municipalities, which allows us to establish the relationship:

$$\text{phone cell towers} \rightarrow \text{list of municipalities},$$

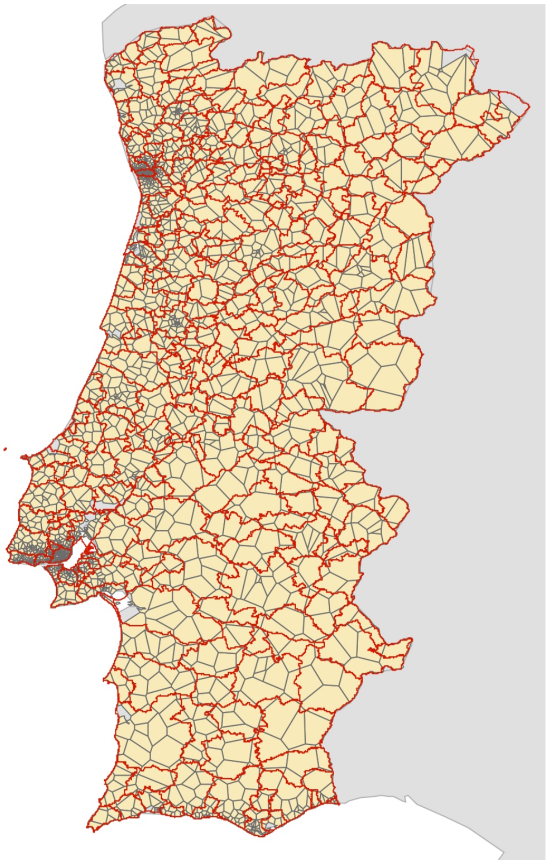

Figure S1: Mobile phone tower cells of Portugal (in grey) excluding those located in the Azores and Madeira Islands. Red lines indicate the boundaries of the Portuguese municipalities (concelhos).

where each cell c has a relative weight $w_{c,M}$ assigned for each municipality M that partially contains it. The relative weights are normalized to 1:

$$\sum_{M} w_{c,M} = 1. \tag{1}$$

Given a commuting flux T_{st} going from cell s to cell t, this translates into a contribution for the commuting flux T_{IJ} between municipalities I and J as follows:

$$T_{IJ} += T_{st} \cdot w_{s,I} \cdot w_{t,J} \tag{2}$$

where $s \in I$ and $t \in J$. If $I = J$ the contribution goes to the internal selfloop of I, $SL(I)$.

We explored different definitions for the weights $w_{c,M}$ based on the population distribution or the area covered by each portion of cell. Intuitively, a large fragmentation of mobile phones

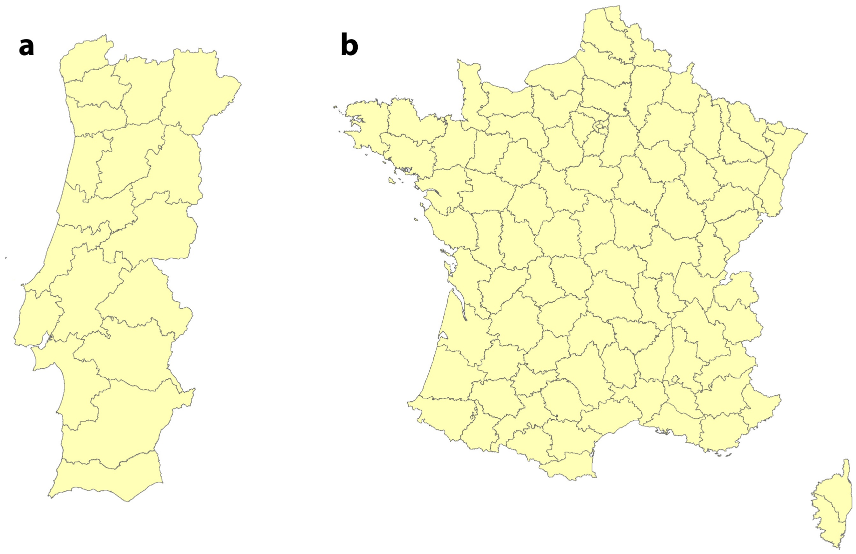

Figure S2: Maps of the Portuguese districts (panel a) and the French departments (panel b).

towers among several census areas leads to a large fragmentation of the resulting coarse-grained network, since the relative weights $w_{c,M}$ can have very small but non-zero values. Such effect is more evident when the geographic resolution of the administrative units is comparable to the cell resolution (such as in Portugal). In that case, the coarse-grained commuting network displays a large number of edges with weights smaller than 1. Removing those edges, however, would imply removing a significant total number of commuters from the system.

Eventually, we find that the best definition of $w_{c,M}$ which preserves the total number of commuters in the network is the following:

$$w_{c,M} = \begin{cases} 1, & \text{if tower coordinates} \in M \\ 0, & \text{if tower coordinates} \notin M \end{cases} \tag{3}$$

which assigns each tower to a single municipality according to the coordinates of the tower. We employ such definition for all the analyses presented in the main text.

2 Additional Results

2.1 Results for lower resolutions

In order to test the effects of spatial resolution on our results, we aggregated all the commuting networks of Portugal and France at the level of districts and departments, respectively. The geographic resolution of these administrative units in the two countries is shown in Figure S2. In France we excluded overseas departments from our analysis, while in Portugal we excluded the Azoras and Madeira islands.

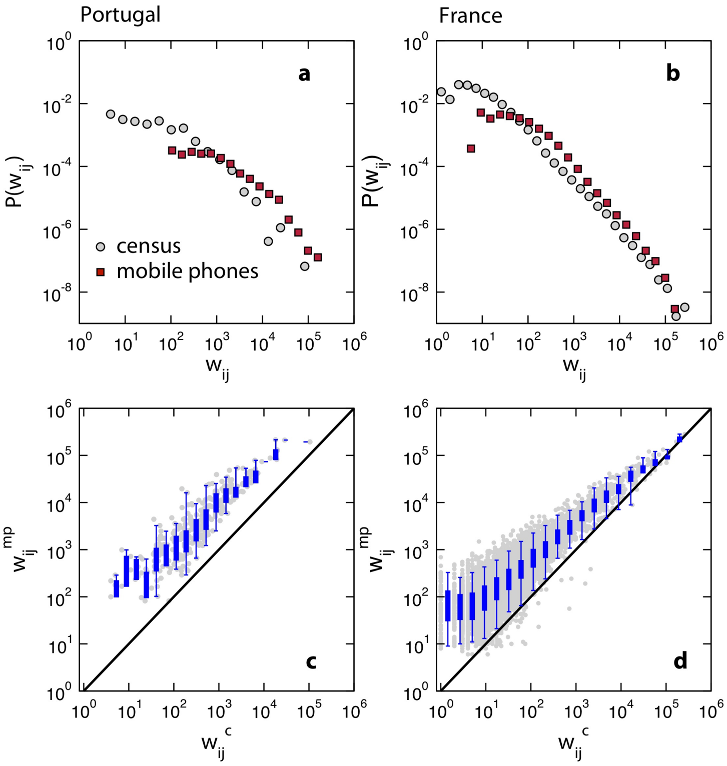

Figure S3: Top: probability density distributions of the weights (w_{ij}) of the census commuting network (grey) and the mobile phone commuting network (red) in Portugal (panel a), and France (panel b) at the level of districts and departments, respectively. Bottom: comparing weights in the mobile phone network (w^{mp}) and weights in the census networks (w^{c}). Grey points are scatter plot for each pair of subdivisions. Box plots indicate the 95% reference range of values within a bin.

Following the analysis presented in the main text, in Figure S3 we report the comparison of the weight distributions in the census network and the mobile phones network of Portugal, at the level of districts, and France, at the level of departments. Figure S4 shows the side-by-side comparison of the weights in the two networks, as a function of distance, population of origin and population of destination.

Figure S5 shows the results of epidemic simulations performed on the aggregated commuting networks at the level of Portuguese districts and French departments. As in the main text, we consider three values of the basic reproduction number, three mobility networks and three initial seeds of the outbreak. Seeds are chosen to match the same geographic locations considered in the main text, at a finer geographic resolution.

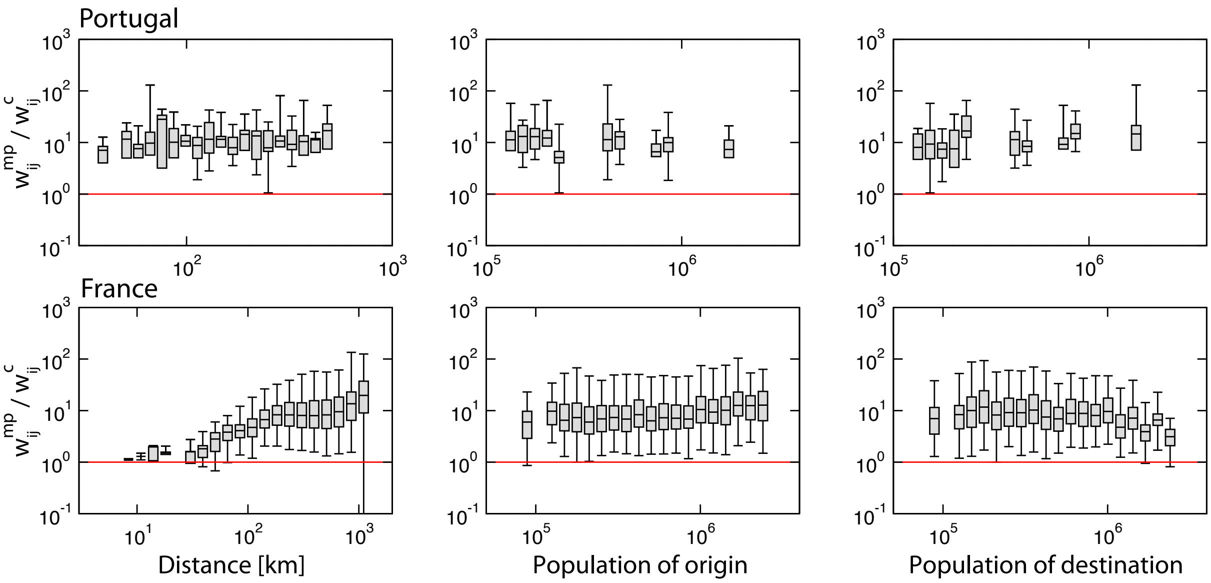

Figure S4: Panels show the ratio between the weights of the mobile phone networks w^{mp} and the census networks w^c in Portugal, at the level of districts, and in France, at the level of departments, as function of the Euclidean distance between nodes (left panels), the population of origin (middle panels) and the population of destination (right panels). The solid line indicates the unit value.

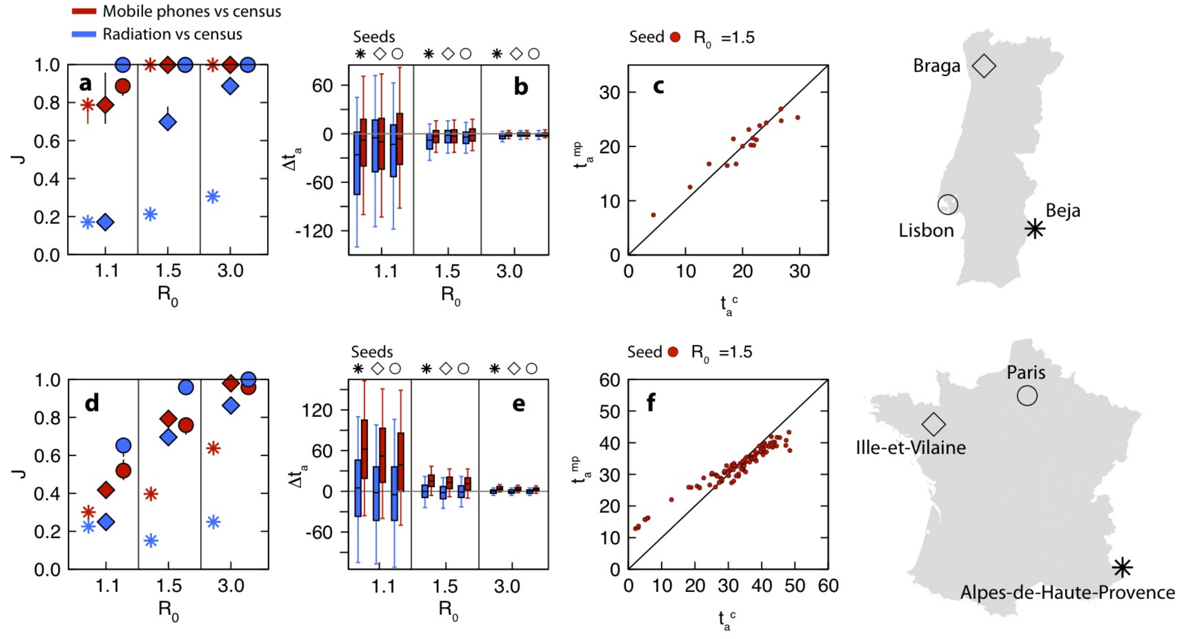

Figure S5: Comparing the epidemic behavior on the census network and two proxy networks, mobile phone (red symbols) and radiation model (blue symbols), for Portuguese districts (top panels), and French departments (bottom). Panels a, d: Jaccard similarity index measured between the epidemic infection tree of the census network and the infection tree of the proxy network, for three values of the basic reproduction number R_0. Each symbol corresponds to a different initial infection seed, displayed on the map. Panels b, e: differences between the arrival times in the census network and in the proxy network, for different values of R_0 and infection seed. Box plots indicate the 90% reference range, measured on all the network nodes. Panels c, f: comparing the arrival times in the mobile phone network t_a^{mp} with those in the census network t_a^c, for $R_0 = 1.5$ and the epidemic starting from the capital city. Red points are scatter plot for each node of the network and we subtracted the average systematic difference $\langle \Delta t_a \rangle$ from each t_a^{mp}.

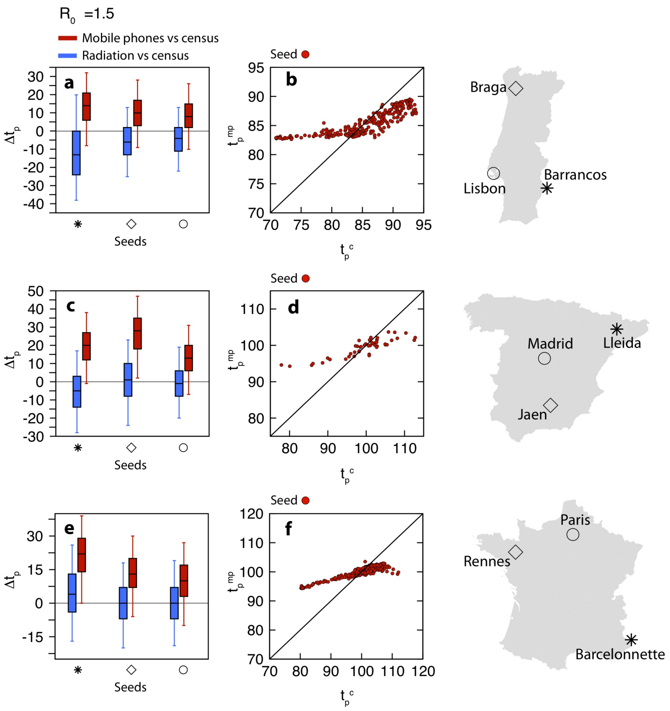

Figure S6: Comparing the epidemic peak times on the census network and two proxy networks, mobile phone (red symbols) and radiation model (blue symbols), for Portugal (top panels), Spain (middle) and French (bottom). For all simulations, $R_0 = 1.5$. Panels a, c and e: differences between the peak times in the census network and in the proxy network for different infection seeds, as shown in the maps. Box plots indicate the 90% reference range, measured on all the network nodes. Panels b, d and f: comparing the peak times in the mobile phone network t_p^{mp} with those in the census network t_p^c , for epidemics starting from the capital city. Red points are scatter plot for each node of the network and we subtracted the average systematic difference $\langle \Delta t_p \rangle$ from each t_p^{mp}.

2.2 Results on epidemic peak times

Here, we report the additional comparison between the epidemic peak times of simulations based on the census network and the proxy networks (mobile phones and radiation). Figure S6 shows the differences between the peak times in the census network and in the proxy network for different infection seeds in the three countries, chosen as in the main text. The geographic resolution is the same of the main text (Portuguese municipalities, Spanish provinces, French districts).

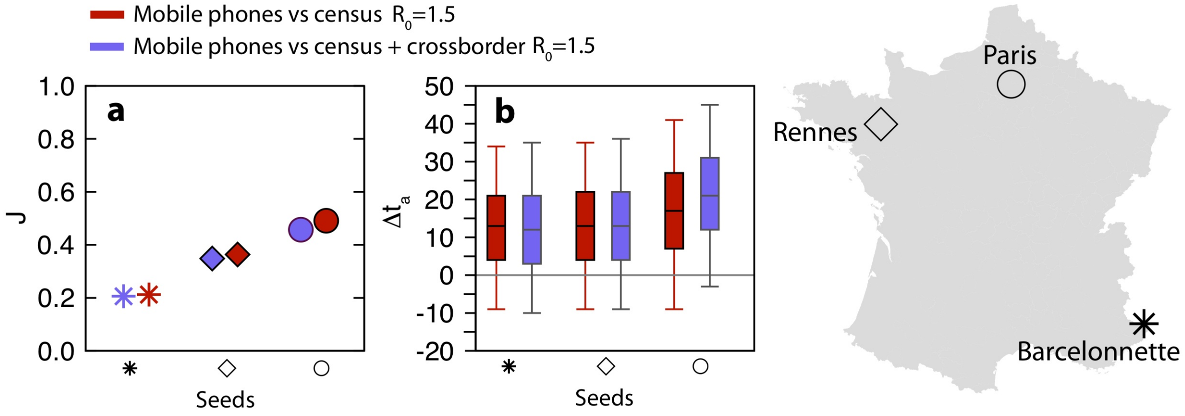

Figure S7: Comparing the epidemic behavior on the mobile phone network and two versions of the census network: the one without cross-border commuting (red symbols) and the one that includes cross-border commuters (purple symbols). Panel a: Jaccard similarity index measured between the epidemic infection tree of the census networks and the infection tree of the mobile phone network, for $R_0 = 1.5$. Each symbol corresponds to a different initial infection seed, displayed on the map. Panel b: differences between the arrival times in the census network and in the proxy network, for $R_0 = 1.5$ and infection seed. Box plots indicate the 90% reference range, measured on all the network nodes.

3 Sensitivity analysis

3.1 Cross-border commuting

As explained in the main text, in our study we disregarded cross-border commuting fluxes. Here, we provide additional analyses to support our choice. The main reasons to remove cross-border connections are:

1. cross-border commuting can not be extracted from mobile phones and it is not well captured by the radiation model;

2. cross-border commuters represent a tiny fraction of the total in the countries under study.

In particular, we found that cross-border commuting represents less than 1% of the total commuting fluxes in Portugal and Spain. In France, cross-border commuting is a more relevant phenomenon: in some border regions - especially in the proximity of Switzerland and Monaco - the number of outgoing cross-border commuters may exceed the number of the commuters who travel within the country.

In order to evaluate the impact of removing such connections with abroad, we examined the 2007 census commuting network of France at the level of districts. We considered a revised version of the network where we included the cross-border connections, as reported by the official census, adding a total of 305, 756 cross-border commuters traveling abroad to 29 different

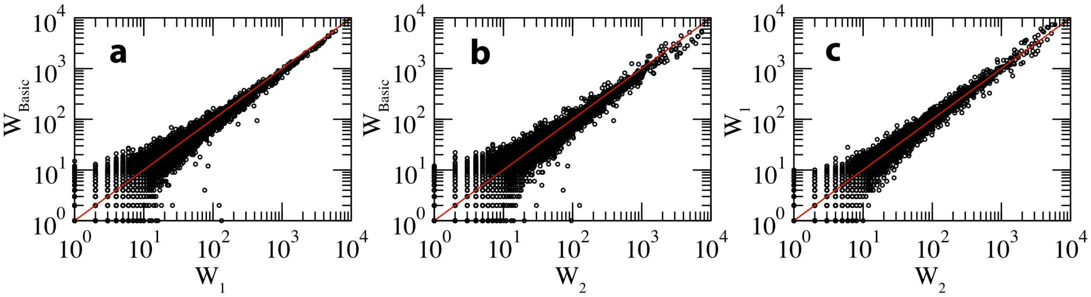

Figure S8: Comparing the weights of mobile phone networks based on different definitions of a user's home and workplace in Portugal, at the level of municipalities. Weights are normalized according to the basic normalization (see main text) From left to right: comparing the weights of the basic definition against definition 1 (panel a), basic definition against definition 2 (panel b) and definition 1 against definition 2 (panel c). Black points are scatter plot for each link in the intersection of the two networks. The identity line is shown in red.

destinations along $1,500$ new links. More precisely, all the French cross-border commuters are directed to:

- 3 States (Länder) in Germany;
- 9 Provinces in Belgium;
- 3 Cantons in Luxembourg;
- 13 Cantons in Switzerland;
- the Principality of Monaco.

First, we compared the results of simulated epidemics based on the census network without cross-border commuting and including cross-border commuting to test the net effect of adding new nodes and connections. We seeded the epidemics in Paris, Rennes and Barcelonnette with $R_0 = 1.5$. For all the seeds, the observed median difference between the arrival times in the two networks is zero. The associated reference range does not show any significant difference between the two systems.

Eventually, we also compared the results of simulated epidemics based on the census network with cross-border commuting and the mobile phone network. As shown in Figure S7, results do not show any relevant deviation from those obtained without including cross-border commuting.

3.2 Refined definitions of workplace and residence from mobile phone activity data

In order to identify each user's residence and workplace we generally assumed that they correspond to the most and second most visited location (tower cell), respectively, in terms of the

country	administrative level	$\mathbf{w_{ij}}$		incoming commuters	
		Lin	*Spearman*	*Lin*	*Spearman*
Portugal	municipalities	0.69	0.62	0.86	0.91
Spain	provinces	0.73	0.75	0.69	0.55
France	districts	0.74	0.65	0.95	0.95

Table S2: Statistical comparison between census and mobile phone data with a refined normalization. Values of the Lins concordance coefficients after a log transformation of variables, and Spearmans coefficient measured between the mobile phone network and the census network for the weights (w_{ij}) and the nodes' total fluxes of incoming commuters. Rows correspond to different countries.

total number of calls placed. In order to test the impact of our assumption, we also investigated the effects of imposing additional constraints to refine the basic definition of the main text and we tested different approaches on one of our datasets. In detail, we compared the commuting networks extracted from the Portuguese mobile phone dataset by defining each user's home and workplace in three ways:

- **Basic definition**: the first and second most visited locations during the whole day, with no other constraints, as defined in the main text;
- **Definition 1**: the most visited location during night hours (midnight - 6am) and the most visited location during the rest of the day;
- **Definition 2**: the most visited location during night hours (6pm - 6am) and the most visited location during working hours (6am - 6 pm) excluding weekends;

where definitions 1 and 2 represent our sensitivity analysis.

The resulting commuting networks all display a significant overlap in terms of connections (Jaccard index > 0.55) and a strong correlation between the commuting flows, as displayed in Figure S8. Furthermore, for all the three networks, the intersection with the census network accounts for more than 97% of the commuters in both networks. We conclude that the results of the network extraction and subsequent aggregation are robust against changes in the definition of home and workplace of a user.

3.3 Refined normalization of mobile phones data

Here, we report some of the results of the analyses based on a refined normalization of the mobile phones networks. In detail, we normalize the weights of the mobile phone networks by imposing the total number of commuters who live in a given administrative unit (Portuguese municipality, Spanish province or French district) to be equal to the number of commuters reported by census. We label the weights in the mobile phone networks after the refined normalization as w^{mp*}, while for the basic normalization reported in the main text we use the notation w^{mp}.

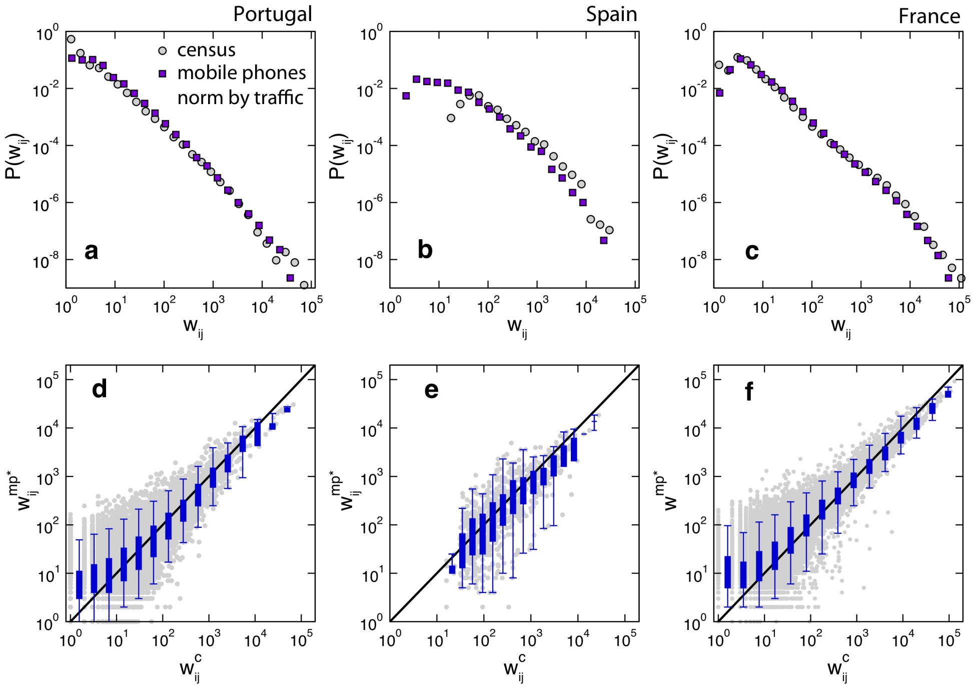

Figure S9: Comparing the weights of the census networks and the mobile phone networks with a refined normalization. Top: probability density distributions of the weights of the census commuting network (grey) and the mobile phone commuting network (purple) in Portugal (panel a), Spain (panel b) and France (panel c). Bottom: comparing weights in the mobile phone network (w^{mp*}) and weights in the census networks (w^c). Grey points are scatter plot for each pair of subdivisions. Box plots indicate the 95% reference range of values within a bin.

Figure S9 displays the comparison between the weight distributions in the census network and the mobile phone network with the refined normalization. As shown in Table S2, the correlation between weights and incoming traffic in the two datasets is strong and generally higher than the correlation measured with the basic normalization (Table 2 of the main text). We do not show the correlation values for a node's outgoing traffic because large values are a trivial effect of the refined normalization.

3.4 Topological distance between regions

In the main text and previous sections, we showed how the ratio w^{mp}/w^c varies as a function of the Euclidean distance between connected nodes. Euclidean distance is the quantity of interest for many theoretical models of mobility, however, a comparison between countries may be hindered by the fact that the geographic resolution is not homogeneous across them and the Euclidean distance between centroids is not fully representative. Using the Euclidean distance, neighboring regions may appear to be far only because they cover a wide area.

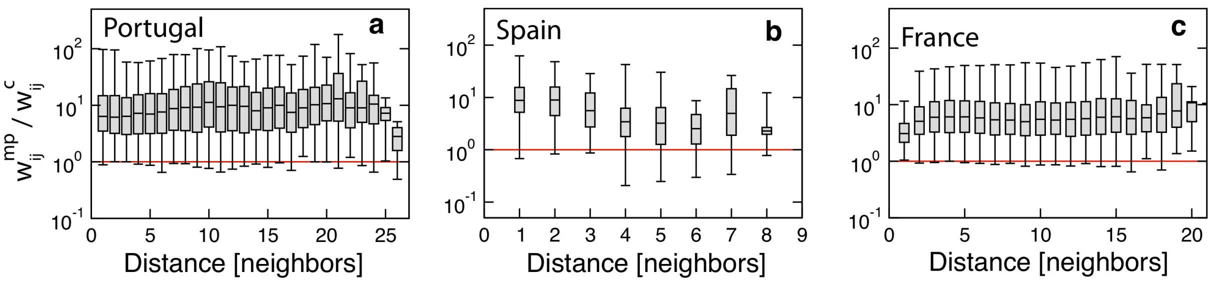

Figure S10: Panels show the ratio between the weights of the mobile phone networks $w^{mp}$and the census networks w^c in Portugal (a), Spain (b) and France (c), as function of the hop distance between administrative regions (Portuguese municipalities, Spanish provinces and French districts).

In order to test the effects of using the Euclidean distance between centroids, we considered the *hop distance* between administrative units defined by a neighbor joining approach. With this approach, neighboring regions have distance 1, neighbors of neighbors have distance 2 and so on.

In Figure S10, we show the ratio w^{mp}/w^c as a function of the hop distance in the three countries under study. The difference between weights does not show any significant correlation with the hop distance, indicating that the results observed using the Euclidean distance may be mainly due to the adopted geographic resolution.

4 Simulation algorithm

Here, we report the pseudo-code of the algorithm used to simulate the SIR dynamics on the metapopulation commuting network. Each node of the network has a resident population $N_i = \sum_i N_{ij}$, where N_{ij} is a matrix that contains the number of individuals who live in node i and work in node j. Individuals are labeled according to their health status and divided into: S_{ij}, I_{ij} and R_{ij}. The entries of the matrices S, I and R are initialized by setting $S_{ij} = N_{ij}$ for every i and j but in the initial seed s. Therefore, at the beginning of the simulation the only non-zero entry of the matrix I will be $I_{ss} = 10$.

Each simulation time step is divided into two parts: work time and home time assumed to have equal length (12 hours each). During work time, the force of infection in node i is calculated as:

$$\lambda_i^{work} = \frac{\beta}{2} \frac{I_{ii} + \sum_j I_{ji}}{N_{ii} + \sum_j N_{ji}}, \tag{4}$$

where β is the daily transmissibility and the factor 1/2 takes into account that we are considering half a day. New infected individuals among those who work in i are extracted using a random binomial sampling:

$$\Delta(S_{ij} \to I_{ij}) = Binom(S_{ij}, \lambda_i^{work}) \,. \tag{5}$$

Analogously, during home time, the force of infection in node i is calculated as:

$$\lambda_i^{home} = \frac{\beta}{2} \frac{I_{ii} + \sum_j I_{ij}}{N_{ii} + \sum_j N_{ij}} \,. \tag{6}$$

New infected individuals among those who live in i are extracted using a random binomial sampling:

$$\Delta(S_{ij} \to I_{ij}) = Binom(S_{ij}, \lambda_i^{home}) \,. \tag{7}$$

Recovery transitions happen both during home and work time, with constant probability $\mu/2$, where μ is the daily recovery rate.

Algorithm 1 SIR dynamics in the metapopulation system

```
for all run r do

   read population database
   initialize epidemic variables

   for all timestep t do

      {Work time}
      for all node i do
         evaluate the force of infection at work using Eq.4
         extract infections using random binomials with probability given by λ_i^work
         extract recoveries using random binomials with probability equal to μ/2
         update compartments
      end for

      {Home time}
      for all node i do
         evaluate the force of infection at home using Eq.6
         extract transitions using random binomials with probability given by λ_i^home
         extract recoveries using random binomials with probability equal to μ/2
         update compartments
      end for

   end for
   print output variables
end for
```
